# Disease burden and associated risk factors for metabolic syndrome among adults in Ethiopia

**DOI:** 10.1186/s12872-019-1201-5

**Published:** 2019-10-26

**Authors:** Samrawit Solomon, Wudeneh Mulugeta

**Affiliations:** 1 Department of Public Health, St. Paul’s Hospital Millennium Medical College, Addis Ababa, Ethiopia; 2 Department of Medicine, Harvard Medical School, Cambridge Health Alliance, 454 Broadway; Revere, Cambridge, MA USA

## Abstract

**Background:**

Metabolic Syndrome (MetS) and Non-communicable diseases (NCDs) are alarmingly increasing in low-income countries. Yet, very limited is known about the prevalence and risk factors associated with MetS in Ethiopia.

**Methods:**

A cross-sectional study was conducted among adult outpatients (*N* = 325) at St. Paul’s Hospital Millennium Medical College in Addis Ababa, Ethiopia. The study was conducted in accordance with STEPwise approach of the World Health Organization. MetS was defined using modified National Cholesterol Education Program’s Adult Treatment Panel III criteria. Univariate and multivariate analyses were performed.

**Results:**

The overall prevalence of MetS was 20.3%. Among the 325 participants, 76.9% had at least one MetS components. Reduced high-density lipoprotein cholesterol was the most common MetS component at 48.6%, followed by elevated blood pressure at 36.3%, and elevated fasting glucose at 32.6%. Older age (odds ratio [OR] = 4.15; 95% confidence interval [CI] = 1.43–12.04), Amhara ethnicity (OR = 2.36; 95%CI = 1.14–4.88), overweight status (OR = 2.21; 95%CI = 1.03–4.71), higher income (OR = 3.31; 95%CI = 1.11–9.84) and higher education levels (OR = 2.19; 95%CI = 1.05–4.59) were risk factors for MetS.

**Conclusion:**

The disease burden of MetS among Ethiopians is high, and is associated with age, weight, income, education and ethnicity. Comprehensive screening and assessment of MetS is needed along with effective preventive and treatment strategies in low-income countries, such as Ethiopia.

## Background

Non-communicable diseases (NCDs) are the leading causes of death globally, killing more people each year than all other causes combined [1]. Nearly 85% of the global premature deaths associated with NCDs occur in low- and middle-income countries [1]. If the current growing burden of NCDs continues, the cumulative loss to global economy has been estimated to reach $47 trillion by 2030 [2]. Currently, one of the leading global public-health challenges is metabolic syndrome (MetS), which includes abdominal obesity, dyslipidemia, hyperglycemia, and hypertension. MetS has been linked to the risk of developing cardiovascular disease (CVD) and type 2 diabetes mellitus [3]. Visceral adiposity and insulin resistance are thought to be the underlying mechanisms for MetS [3].

Most of the studies on MetS have been conducted in North America, Europe, and Asia [4–6]. As a result, very limited is known about the prevalence and risk factors of metabolic syndrome among sub-Saharan African population. The few studies conducted in sub-Saharan Africa show the prevalence of metabolic syndrome is rapidly approaching that of the developed nations [7, 8]. This could be because of adaptations of unhealthy Western diet and lifestyle, as well as tobacco use, and use of anti-HIV drugs in those areas [9, 10]. Recently, the rapid economic growth along with the aging population and the sedentary lifestyle in sub-Saharan countries like Ethiopia has increased the NCDs epidemics [8, 11, 12].

Although there have been few other studies in sub-Saharan Africa, this is one of the first studies to examine disease burden and risk factors for Metabolic Syndrome at a major referral hospital in Ethiopia in a general outpatient setting [13, 14]. Given this gap in the literature, and given the importance in developing health promotion and disease prevention programs in low- and middle-income countries, we examined the prevalence and risk factors of metabolic syndrome and its components in Ethiopia.

## Methods

### Study design and participants

This hospital based cross-sectional study was conducted from September 2017 to October 2017 at the outpatient departments at St. Paul’s Hospital Millennium Medical College (SPHMMC) in Addis Ababa, the capital city of Ethiopia. SPHMMC is one of the largest teaching and referral hospitals in the country with inpatient capacity of more than 1000 beds, and an average of 2000 emergency and outpatient clients daily. We investigated patients from outpatient department not in chronic follow-up OPD’s (where patients are known hypertensive, diabetic or other chronic disease follow-up patients) because those are patients who presented to St Paul’s hospital millennium medical college with different types of complaints. The inclusion criteria were all eligible adult patients coming to outpatient department at SPHMMC during the study period. The exclusion criteria were patients at departments other than the outpatient department at SPHMMC, such those who were getting care inpatient, and those patients who did not fast overnight for at least 8 h. Patients unable or unwilling to provide informed consent to participate in the study in Amharic or Oromifa were also excluded. However, all of the potential study participants approached spoke Amharic and/or Oromifa languages. The study was conducted in accordance with a modified STEPwise approach to non-communicable disease risk factor surveillance of the World Health Organization (WHO), which includes questionnaire, physical and biochemical measurements [15]. Ethical clearance was obtained from Institutional Review Board of SPHMMC, Addis Ababa, Ethiopia and an official letter of permission was obtained from the research directorate. Patients’ confidentiality was kept throughout the study and names of individuals was not included in the study at any phase. Written consent was obtained before inclusion into the study after announcing the details of the study including the benefits and risks.

### Data collection and variable specification

Participants were interviewed by a trained health officer and a nurse using the modified WHO STEPwise questionnaire. The questionnaire was in English, translated into Amharic and Oromifa, which are common languages in Ethiopia, and then translated back into English.

Trained staff health officers collected blood samples and standard anthropometric measurements. Manual blood pressure (BP) was measured with participants sitting, after resting for at least 5 min. The waist circumference was taken at the borderline between the lower boundary of the last palpable rib and the top of the iliac crest. Body mass index (BMI) was calculated by dividing weight in kilograms by height squared in meters (kg/m2) with regular monitoring and adjustment of the beam-balance. Weight was categorized using the National Heart, Lung, and Blood Institute’s classification system.

After an 8-h overnight fasting, blood specimen was collected from every participant to determine the fasting blood sugars and lipid profiles in the college’s clinical chemistry laboratory. Triglyceride (TG) concentrations were measured by standard enzymatic assays using glycerol phosphate oxidase method. High-density lipoprotein cholesterol (HDL-C) was determined after sample pretreatment with a precipitating reagent and centrifugation. Participants’ fasting blood glucose (FBG) were determined using glucose oxidase method. Normal and pathological quality control materials were run every day to detect any analytical errors and validate the laboratory values. Standard operating procedures for all the quality assurance phases were utilized.

Determination of Metabolic Syndrome (MetS): Metabolic Syndrome was defined using the modified National Cholesterol Education Program’s Adult Treatment Panel III criteria (NCEP ATP III), and a joint definition that was agreed upon by several organizations (International Diabetes Federation Task Force on Epidemiology and Prevention; National Heart, Lung, and Blood Institute; American Heart Association; World Heart Federation; International Atherosclerosis Society; and International Association for the Study of Obesity) [16, 17]. In accordance with the NCEP ATP III criteria, subjects were classified as having Met S if participants have three or more of the following risk factors: (1) Abdominal obesity (waist circumference > 102 cm in men and > 88 cm in women) (2) Elevated triglyceride (TG ≥150 mg/dL) (3) Reduced HDL-C (< 40 mg/dL in men and < 50 mg/dL in women) or drug treatment (4) High blood pressure (≥130/85 mmHg) or drug treatment and (5) fasting blood glucose (≥110 mg/dL) or drug treatment [16, 17]. In accordance with the International Diabetes Federation (IDF) criteria, subjects were classified as having MetS if a participant has abdominal obesity (defined as waist circumference of ≥94 cm for men and ≥ 80 cm women) plus two of any of the following risk factors: (1) Elevated triglyceride (≥150 mg/dL) (2) Reduced HDL-C (< 40 mg/dL in men and < 50 mg/dL in women) or drug treatment (3) Elevated blood pressure (systolic BP ≥130 or diastolic BP ≥85 mmHg) or drug treatment (4) Elevated fasting blood glucose (≥100 mg/dL) or drug treatment [18].

### Statistical analyses

Frequency distributions of socio-demographic and behavioral characteristics of study participants were examined. The overall prevalence and prevalence estimates of metabolic syndrome across socio-economic groups were calculated separately based on gender. Chi-square tests were used to assess differences in the distributions of categorical variables.

Univariate and multivariate binary logistic regression analyses were performed to examine factors associated with metabolic syndrome. The regression models were built with metabolic syndrome as the outcome variable after adjusting for covariates. Covariates were included in the final regression model using *p*-value < 0.2 as a cut-off, as well as based on priori and our conceptual framework. Adjusted odds ratios (AOR) with 95% confidence intervals (95% CI) were used to determine the magnitude of associations among metabolic syndrome and various potential risk factors. Two-tailed statistical significance was assessed at α < 0.05. All the analyses were conducted in 2018 using statistical software SAS 9.4 (SAS Institute Inc., Cary, NC).

## Results

Basic demographic characteristics of the participants are provided in Table 1. Out of the 325 study participants, 155 (47.7%) were men and 170 (52.3%) were women. Nearly a quarter (24.6%) of the participants was aged 51 years or older, whilst 33.2% were younger than 30 years of age. The majority of participants reported to be married (62.8%) and reside in Addis Ababa (63.6%). Most participants reported to be Oromo (44.7%) in ethnicity with education levels of primary school or less (59.7%) and non-government employment status (48.0%). The prevalence of underweight, overweight, and obese were 10.4, 18.8, and 3.3%, respectively.

**Table 1:** Basic demographic characteristics of adult outpatients (*N* = 325) at St. Paul’s Hospital Millennium Medical College in Addis Ababa, Ethiopia

Variable		Total *N* = 325 (%)	Men *N* = 155 (%)	Women *N* = 170 (%)	*p-value*
Age, years	< 30	108 (33.2)	43 (27.7)	65 (38.2)	*0.013*
	30–50	137 (42.2)	63 (40.7)	74 (43.5)	
	≥51	80 (24.6)	49 (31.6)	31 (18.2)	
Region	Addis Ababa	199 (63.6)	97 (63.4)	102 (63.8)	*0.646*
	Oromia	78 (24.9)	36 (23.5)	42 (26.4)	
	Others	36 (11.5)	20 (13.1)	16 (10.0)	
Ethnicity	Oromo	142 (44.7)	70 (46.1)	72 (43.4)	*0.851*
	Amhara	103 (32.4)	47 (30.9)	56 (33.7)	
	Others	73 (22.9)	35 (23.0)	38 (22.9)	
Marital status	Single	77 (23.7)	43 (27.7)	34 (20.0)	*0.0004*
	Married	204 (62.8)	103 (66.5)	101 (59.4)	
	Divorced/ widowed	44 (13.5)	9 (5.8)	35 (20.6)	
Income, birr	< 2000	44 (36.7)	19 (30.7)	25 (43.1)	*0.322*
	2000–4000	44 (36.7)	26 (41.9)	18 (31.0)	
	≥4000	32 (26.7)	17 (27.4)	15 (25.9)	
Education	Primary schools or less	178 (59.7)	78 (53.8)	100 (65.4)	*0.114*
	Secondary school or more	120 (40.3)	67 (46.2)	53 (34.6)	
Occupation	Government employees	50 (15.4)	25 (16.3)	25 (14.7)	*< 0.0001*
	Other employees/ students	156 (48.0)	93 (60.0)	63 (37.1)	
	Unemployed	119 (36.6)	37 (23.9)	82 (48.2)	
Weight, BMI in kg/m2	Underweight (< 18.5)	32 (10.4)	13 (8.7)	19 (12.0)	*0.397*
	Normal (18.5–24.9)	208 (67.5)	107 (71.3)	101 (63.9)	
	Overweight (25.0–29.9)	58 (18.8)	27 (18.0)	31 (19.6)	
	Obese (≥30.0)	10 (3.3)	3 (2.0)	7 (4.4)	

*BMI* body mass index in kg/m2

Table 2 shows behavioral and lifestyle characteristics of the study population. Alcohol, Khat, and tobacco use were reported by 32.6, 10.3, and 3.7% of the participants, respectively. Approximately a third (33.6%) of the participants reported a moderate or vigorous level of physical activity at work, while only 4.9% reported regular exercise or leisure physical activity. However, the majority (60.3%) used walking/ bicycle for transportation. Most (71.0%) reported adding salt before or during eating meals, and only 1.5% reported eating 5 or more servings of fruits and vegetables a day.

**Table 2:** Behavioral and lifestyle characteristics of adult outpatients (*N* = 325) at St. Paul’s Hospital Millennium Medical College in Addis Ababa, Ethiopia

Variable		Total *N* = 325 (%)	Men *N* = 155 (%)	Women *N* = 170 (%)	*p-value*
Alcohol use		106 (32.6)	63 (40.7)	43 (25.3)	*0.003*
Tobacco use		12 (3.7)	12 (7.7)	0 (0.0)	*0.0002*
Khat use		33 (10.3)	30 (19.5)	3 (1.8)	*< 0.0001*
Physical activity at work		105 (33.6)	49 (33.1)	56 (33.9)	*0.876*
Number of days with physical activity at work	< 5 days/week	261 (80.3)	118 (76.1)	143 (84.1)	*0.071*
	≥5 days/week	64 (19.7)	37 (23.9)	27 (15.9)	
Doing exercise / leisure physical activity		16 (4.9)	9 (5.9)	7 (4.1)	*0.473*
Walking/ bicycle for transportation		194 (60.3)	92 (60.1)	102 (60.4)	*0.967*
Adding salt before or as eating		230 (71.0)	108 (70.1)	122 (71.7)	*0.746*
Type of oil used	None	129 (39.9)	58 (37.9)	71 (41.8)	*0.525*
	Vegetable oil	133 (41.2)	68 (44.4)	65 (38.2)	
	Other oil/ fat	61 (18.9)	27 (17.7)	34 (20.0)	
Number of days fruits or vegetables were eaten	≥5 days/week	113 (34.8)	54 (34.8)	59 (34.7)	*0.988*
	2–4 days/week	125 (38.5)	59 (38.1)	66 (38.8)	
	< 2 days/week	87 (26.8)	42 (27.1)	45 (26.5)	
Number of fruits or vegetable servings	≥5 servings/day	5 (1.5)	3 (1.9)	2 (1.2)	*0.579*
	< 5 servings/day	320 (98.5)	152 (98.1)	168 (98.8)	

Alcohol use in the past year; current tobacco use; ever used Khat; physical activities: moderate or vigorous work or leisure/ recreational activities/ exercise; adding salt always, often or sometimes vs rarely or never.

Table 3 shows frequency of Metabolic Syndrome (MetS) and MetS components. The prevalence of MetS according to the modified NCEP-ATP III criteria was 20.3% (18.1% among men and 22.4% among women). The most common MetS component was reduced high-density lipoprotein cholesterol (HDL-C) at 48.6%, followed by elevated blood pressure at 36.3%, and elevated fasting glucose at 32.6%. Nearly a quarter (24.0%) of participants had elevated Triglyceride levels, whilst 10.5% had abdominal obesity according to the modified NCEP-ATP III criteria.

**Table 3:** Frequency of metabolic syndrome (MetS) and components among adult outpatients (*N* = 325) at St. Paul’s Hospital Millennium Medical College in Addis Ababa, Ethiopia

Parameters	Total *N* = 325 (%)	Men *N* = 155 (%)	Women *N* = 170 (%)	*p-value*
Metabolic syndrome by NCEP-ATP III	66 (20.3)	28 (18.1)	38 (22.4)	*0.337*
Metabolic syndrome by IDF	28 (8.6)	3 (1.9)	25 (14.7)	*< 0.001*
Abdominal obesity by NCEP-ATP III	34 (10.5)	2 (1.3)	32 (18.8)	*< 0.001*
Abdominal obesity by IDF	63 (19.4)	4 (2.6)	59 (34.7)	*< 0.001*
Elevated fasting glucose	106 (32.6)	57 (36.8)	49 (28.8)	*0.126*
Elevated Triglycerides	78 (24.0)	40 (25.8)	38 (22.4)	*0.467*
Elevated blood pressure	118 (36.3)	65 (41.9)	53 (31.2)	*0.044*
Reduced HDL-C	158 (48.6)	64 (41.3)	94 (55.3)	*0.012*

NCEP ATP III: modified National Cholesterol Education Program’s Adult Treatment Panel III criteria; IDF: International Diabetes Federation; HDL-C: High-density lipoprotein cholesterol

Table 4 shows 76.9% of the participants had at least one MetS components, 46.8% had at least two MetS components, 20.3% had at least three MetS components, 6.8% had at least four MetS components, and 1.2% had all five MetS components in accordance with the modified NCEP-ATP III criteria.

**Table 4:** Number of metabolic syndrome (MetS) components by gender according to the modified NCEP-ATP III criteria among adult outpatients (*N* = 325) at St. Paul’s Hospital Millennium Medical College in Addis Ababa, Ethiopia

Number of MetS components	Total *N* = 325(%)	Men *N* = 155 (%)	Women N = 170 (%)
0	75 (23.1)	35 (22.6)	40 (23.5)
1	98 (30.2)	48 (30.9)	50 (29.4)
2	86 (26.5)	44 (28.4)	42 (24.7)
3	44 (13.5)	22 (14.2)	22 (12.9)
4	18 (5.5)	4 (2.6)	14 (8.2)
5	4 (1.2)	2 (1.3)	2 (1.2)

Factors associated with MetS are summarized in Table 5. Compared to those younger than 30 years of age, those aged 51 years or older (adjusted odds ratio [AOR] = 4.15; 95% confidence interval [CI] = 1.43–12.04), Amhara ethnicity versus Oromo ethnicity (AOR = 2.36; 95%CI = 1.14–4.88), and those who were overweight vs normal weight (AOR = 2.21; 95%CI = 1.03–4.71) were associated with MetS. Study participants with higher income vs low income levels (AOR = 3.31; 95%CI = 1.11–9.84), and higher education level of secondary school or more vs primary school or less (AOR = 2.19; 95%CI = 1.05–4.59) were also associated with MetS. We found those with higher level of education had higher weight status, yet they also engaged in more exercise than those with lower level of education (Appendix).

**Table 5:** Prevalence and factors associated with Metabolic Syndrome (MetS) according to the modified NCEP-ATP III criteria, using logistic regression, among adult outpatients (*N* = 325) at St. Paul’s Hospital Millennium Medical College in Addis Ababa, Ethiopia

Variables		MetS n(%)	Crude OR (95% CI)	*p-value*	Adjusted OR (95% CI)	*p-value*
Sex	Male	28 (18.1)	1.00	*–*	1.00	*–*
	Women	38 (22.4)	1.31 (0.76,2.25)	*0.34*	1.88 (0.93,3.80)	*0.08*
Age, years	< 30	9 (8.3)	1.00	*–*	1.00	*–*
	30–50	29 (21.2)	1.10 (0.64,1.89)	*0.74*	1.88 (0.72,4.87)	*0.19*
	≥51	28 (35.0)	2.93 (1.65,5.21)	*0.0002*	**4.15 (1.43,12.04)**	***0.01***
Ethnicity	Oromo	21 (14.8)	1.00	*–*	1.00	*–*
	Amhara	30 (29.1)	2.12 (1.22,3.70)	*0.01*	**2.36 (1.14,4.88)**	***0.02***
	Others	14 (19.2)	0.91 (0.47,1.76)	*0.79*	1.84 (0.78,4.37)	*0.17*
Marital status	Single	7 (9.1)	1.00	*–*	1.00	*–*
	Married	44 (21.6)	1.24 (0.70,2.19)	*0.46*	1.37 (0.49,3.82)	*0.55*
	Divorced/ widowed	15 (34.1)	2.33 (1.17, 4.67)	*0.02*	1.95 (0.65,6.85)	*0.29*
Income, birr	< 2000	11 (25.0)	1.00	*–*	1.00	*–*
	2000–4000	11 (25.0)	1.37 (0.65,2.88)	*0.41*	2.37 (0.93,6.05)	*0.07*
	≥4000	9 (28.1)	1.62 (0.71,3.69)	*0.25*	**3.31 (1.11,9.84)**	***0.03***
Education	Primary school or less	30 (16.9)	1.00	*–*	1.00	*–*
	Secondary school or more	31 (25.8)	1.72 (0.98,3.03)	*0.06*	**2.19 (1.05,4.59)**	***0.04***
Occupation	Other employees/ students	26 (16.7)	1.00	*–*	1.00	*–*
	Government employees	10 (20.0)	0.98 (0.46,2.08)	*0.95*	0.46 (0.16,1.31)	*0.14*
	Unemployed	30 (25.2)	1.59 (0.92,2.75)	*0.09*	1.18 (0.58,2.40)	*0.64*
Weight, BMI in kg/m2	Normal (18.5–24.9)	33 (15.9)	1.00	*–*	1.00	*–*
	Underweight(< 18.5)	4 (12.5)	0.53 (0.18,1.57)	*0.25*	0.82 (0.23,2.86)	*0.75*
	Overweight (25.0–29.9)	21 (36.2)	2.80 (1.50,5.23)	*0.001*	**2.21 (1.03,4.71)**	***0.04***
	Obese (≥30.0)	4 (40.0)	2.72 (0.75,9.94)	*0.13*	1.65 (0.35,7.90)	*0.53*
Adding salt before or during eating	No	27 (28.7)	1.00	*–*	1.00	*–*
	Yes	38 (16.5)	0.49 (0.28,0.87)	*0.01*	0.59 (0.31,1.13)	*0.11*
Doing exercise	Yes	1 (6.3)	1.00	*–*	1.00	*–*
	No	64 (20.9)	0.20 (0.03,1.94)	*0.19*	0.18 (0.01,3.95)	*0.28*

*OR* odds ratio, *CI* confidence interval, moderate or vigorous leisure/ recreational activities/ exercise; adding salt always, often or sometimes vs rarely or never

Boldface are variables with *p* value less than 0.05

## Discussion

This study was conducted to assess the burden of metabolic syndrome and to identify associated risk factors among St Paul’s Hospital Millennium Medical College outpatient department in Addis Ababa, Ethiopia. The findings from this study confirm high prevalence of MetS at 20.3% among adults in Ethiopia, according to the modified NCEP-ATP III criteria. The study found the most common MetS component was reduced high-density lipoprotein cholesterol (HDL-C) at 48.6%, followed by elevated blood pressure at 36.3%, and elevated fasting glucose at 32.6%. Study participants with advanced age, higher income and higher education level of secondary school or more had higher risk of MetS. Furthermore, Amhara ethnicity (OR = 2.36; 95%CI = 1.14–4.88), and overweight status (OR = 2.21; 95%CI = 1.03–4.71) were associated with MetS.

The prevalence of MetS according to the modified NCEP-ATP III criteria was a bit lower (20.3%) than that of a study done at Jimma University Teaching Hospital, in Southwest of Ethiopia, which was 26.2% and in this study the prevalence in men and women was 18.1 and 22.4%, respectively, which is also comparable with that of the results from Jimma University where MetS was twice as likely to occur in females as in males [19]. This can be explained because of the similarity in the nature of the study population where they are patients visiting the outpatient department in a referral hospital. The slightly lower prevalence of MetS in our study could be attributed to younger study participants as well as regional differences compared to the study at Jimma University [19]. Other hospital based studies in Ethiopia showed higher prevalence of MetS, but they were conducted solely in hypertensive patients [13, 20]. As expected, the overall prevalence of metabolic syndrome in our study populations was higher than prior community based studies conducted in Addis Ababa and nationally (12.5 and 4.8%, respectively) [14, 21].

The most common MetS component was reduced high-density lipoprotein cholesterol (HDL-C), followed by elevated blood pressure and elevated fasting glucose which is similar with the study in Jimma where hypertension, hyperglycemia, and low HDL-cholesterol are predominant components of Mets but the rate of low density lipoprotein is higher in this study which can be explained by high rate of abdominal obesity and overweight [19]. The study found abdominal obesity was higher among men than women, which is consistent with the findings of other studies and global trends [14, 19, 22].

In our study, compared to those younger than 30 years of age, those aged 51 years or older (adjusted odds ratio [AOR] = 4.15; 95% confidence interval [CI] = 1.43–12.04) and those who were overweight vs normal weight (AOR = 2.21; 95%CI = 1.03–4.71) were associated with MetS. This result is comparable with the national survey done in Ethiopia where individuals aged 65 years and older were at increased risk of developing the metabolic abnormality but lack of physical activity was not associated with MetS in this study as in most of similar studies in Ethiopia [20, 21]. The lack of association between MetS and exercise in our study, as well as other similar studies, is likely due to lack of power in light of the low overall prevalence of exercise or leisure physical activity among the population.

Similar to other studies, we found income and education levels are associated with MetS [13, 20, 23, 24]. Our findings showed MetS was associated with participants with higher income vs low income levels (AOR = 3.31; 95%CI = 1.11–9.84), and higher education level of secondary school or more vs primary school or less (AOR = 2.19; 95%CI = 1.05–4.59). The relationships are likely different between high-income [23] and low-income countries [13, 24]. As those with lower income and education levels in developing countries, likely do more physical and labor intensive types of jobs, while those with higher income and education levels could be less physically active and may also be adapting unhealthy lifestyles [13, 24].

Our study has several strengths which powered to make adequate subgroup comparisons; it is also one of the first studies to examine MetS and its risk factors at a major referral hospital in Ethiopia, including a diverse general outpatient population. Our study has several limitations. First, as a cross-sectional study, there is lack of temporality and causality in the study. Second, social desirability biases may lead to underestimating some of the lifestyle and behavioral questions, such as smoking and alcohol consumptions. Third, as a hospital-based study, generalizability of the study findings to the broader Ethiopian population is limited. Despite these limitations, the study makes significant contribution and fills a substantial gap in the current literature. Further multicenter investigations are needed to understand the underlying reasons and modifiable risk factors behind subgroup differences in more generalizable study settings.

## Conclusions

In conclusion, this study contributes to the substantial gap in the current literature regarding MetS and its risk factors in sub-Saharan Africa. There is significant disease burden of MetS among the general adult outpatients in Ethiopians. MetS risk increases with advanced age, weight status, income, education and certain ethnic groups. Comprehensive screening and assessment of MetS and its modifiable risk factors is needed along with effective preventive and treatment strategies in low-income countries, such as Ethiopia.

## Data Availability

The datasets used and/or analyzed during the current study are available from the corresponding author on reasonable request.

## Appendix

**Table 6:** Income and education levels by weight, physical activity and smoking status of adult outpatients (*N* = 325) at St. Paul’s Hospital Millennium Medical College in Addis Ababa, Ethiopia

BMI category in kg/m2 (%)		< 18.5	18.5–24.9	25.0–29.9	≥30.0	*p-value*
Income, birr	< 2000	69.1	7.1	16.6	7.1	*0.834*
	2000–4000	72.5	7.5	17.5	2.5	
	≥4000	66.6	3.3	26.7	3.3	
Education	Primary schools or less	73.1	10.1	14.9	1.8	*0.010*
	Secondary school or more	57.0	9.7	27.2	6.1	
Regular exercise/ physical activity (%)		Yes		No		*p-value*
Income, birr	< 2000	4.8		95.2		*0.136*
	2000–4000	4.6		95.4		
	≥4000	15.6		84.4		
Education	Primary schools or less	2.8		97.2		*0.031*
	Secondary school or more	8.5		91.5		
Tobacco use (%)		Yes		No		*p-value*
Income, birr	< 2000	4.6		95.4		*0.834*
	2000–4000	2.3		97.7		
	≥4000	3.1		96.9		
Education	Primary schools or less	5.6		94.4		*0.088*
	Secondary school or more	1.7		98.3		

Appendix shows the relationship of weight status by BMI categories and levels of education with physical activity and smoking status among the study participants

## Abbreviations

AOR: Adjusted odds ratios
BMI: Body mass index
CVD: Cardiovascular disease
FBG: Fasting blood glucose
HDL-C: High-density lipoprotein cholesterol
IDF: International diabetes fe*deration
MetS: Metabolic syndrome
NCDs: Non-communicable diseases
NCEP ATP III: National Cholesterol Education Program’s adult treatment panel III criteria
OR: Odds ratio
SPHMMC: St. Paul’s hospital millennium medical college
TG: Triglyceride
WHO: World Health Organization
